# A traditional poly herbal medicine “Le Pana Guliya*”* induces apoptosis in HepG_2_ and HeLa cells but not in CC1 cells: an in vitro assessment

**DOI:** 10.1186/s13065-016-0234-4

**Published:** 2017-01-03

**Authors:** Nekadage Don Amal Wageesha, Preethi Soysa, Keerthi Atthanayake, Muhammad Iqbal Choudhary, Mahinda Ekanayake

**Affiliations:** 1 Department of Biochemistry and Chemistry, Faculty of Medicine, South Asian Institute of Technology and Medicine, Malabe, Sri Lanka; 2 Department of Biochemistry and Molecular Biology, Faculty of Medicine, University of Colombo, Colombo, Sri Lanka; 3 Hussain Ebrahim Jamal Research Institute of Chemistry, Karachi, Pakistan; 4 Department of Biochemistry, Faculty of Science, King Abdulaziz University, Jeddah, Saudi Arabia; 5 No: 9, Moragahapitiya, Balagola, Kengalle, Kandy, Sri Lanka

**Keywords:** Anti-cancer activity, MTT assay, LDH assay, GSH, Rhodamine123, Cytotoxicity

## Abstract

“Le Pana Guliya” (LPG) is a polyherbal formulation which is used to treat different types of cancers in traditional medicine. In this study we describe in vitro efficacy and mechanism of action of LPG on two cancer cell lines (HepG_2_ and HeLa) compared with a normal cell line CC1. The MTT, LDH assays and protein synthesis were used to study antiproliferative activity of LPG while NO synthesis and GSH content were assayed to determine the oxidative stress exerted by LPG. Rhodamine 123 staining, caspase 3 activity, DNA fragmentation and microscopic examination of cells stained with ethidium bromide/acridine orange were used to identify the apoptosis mechanisms associated with LPG. The LPG showed the most potent antiproliferative effect against the proliferation of HepG_2_ and HeLa cells with an EC_50_ value of 2.72 ± 1.36 and 19.03 ± 2.63 µg/mL for MTT assay after 24 h treatment respectively. In contrast, CC1 cells showed an EC_50_ value of 213.07 ± 7.71 µg/mL. Similar results were observed for LDH release. A dose dependent decrease in protein synthesis was shown in both cancer cell types compared to CC1 cells. The reduction of GSH content and elevation of cell survival with exogenous GSH prove that the LPG act via induction of oxidative stress. LPG also stimulates the production of NO and mediates oxidative stress. Rhodamine 123 assay shows the mitochondrial involvement in cell death by depletion of Δψ inducing downstream events in apoptosis. This results in increase in caspase-3 activity eventually DNA fragmentation and LPG induced apoptotic cell death. In conclusion the present study suggested that the LPG exerted an anticancer activity via oxidative stress dependent apoptosis. Therefore present study provides the scientific proof of the traditional knowledge in using LPG as an anticancer agent.

## Background

Plants, marine, and micro-organisms are rich sources of diverse and complex compounds; many of which have potent biological activities that may be beneficial in treating human disease. Early civilizations realized the healing potential of natural products, especially those found in plants. The “Ebers Papyrus”, written in 1500 B.C, outlined the Egyptians usage of 700 drugs, most of which were derived from plants [1]. Over the past two centuries, scientists have employed varying methods of extraction to isolate and identify the “active” compounds of these natural remedies and in doing so uncovered a wealth of chemical diversity. Cancer is characterized by uncontrolled cell division. Almost all cell types can initiate cancerous growth; as such more than 100 malignancies have been recognized [2]. Our understanding on methods of treatment and diagnosis of these diseases has made great strides in the last 50 years in terms of mortality and morbidity; however, many forms of cancers still lack effective treatment options. The ineffectiveness of current chemotherapeutic agents warrants investigations into alternative compounds to improve today’s therapy regimens or to act as a means of chemoprevention. In effort to develop therapeutics for cancer and other diseases, pharmaceutical companies often screen large chemical libraries for potential leads. While screening of these libraries can identify potential leads, compounds synthesized by natural sources also have potential in cancer treatment [3].

The emphasis placed on development of natural products or analogues thereof as therapeutics has proven beneficial. Bark from the Pacific Yew tree (*Taxus brevifolia*), found in the Northwest United States, yields Paclitaxel (Taxol^®^) which is used clinically to treat Kaposi sarcoma, breast, non-small cell lung, and ovarian cancer [4, 5]. In addition, an analogue of paclitaxel, docetaxel (Taxotere^®^), has been developed to treat breast, gastric, prostate, and head and neck cancers [6]. Traditional and indigenous practitioners in Sri Lanka have been treating cancer patients using plant based formulations. In addition to use of a single plant, poly herbal formulations of drugs are intensively used in Sri Lanka. The poly herbal drug named “Le Pana Guliya” (LPG) is a well known drug among the traditional medicinal practitioners which is used to treat various types of cancers. The protocol and the method of preparation are recorded in ‘Ola leaf inscriptions’ belong to their families and passing from one generation to the next.

The mechanism of action of poly herbal drug of this nature with large number of different plant components cannot be revealed through conventional bioassay-guided fractionation. Keeping above in view, the present study was aimed at investigating the cytotoxicity effect of a poly herbal drug “Le Pana Guliya (LPG)” against two different of cancer cell lines compared to the normal healthy cells and reveals the mechanism of action of its cytotoxicity.

## Methods

### Chemicals and equipment

Chemicals needed for cell culture, Folin-Ciocalteu reagent, sodium carbonate (Na_2_CO_3_), aluminum chloride (AlCl_3_), sodium nitrite (NaNO_2_), sodium hydroxide (NaOH) were purchased from Sigma-Aldrich (St Louis, MO63178, USA). TritonX-100 was purchased from Fluka. Tris base was purchased from Promega (Madison, WI 53711–5399, USA). Other chemicals were obtained from Sigma-Aldrich Co (St Louis, MO, USA) unless indicated otherwise. All chemicals used were of analytical grade.

Shimadzu UV 1601 UV visible spectrophotometer (Kyoto, Japan) was used to measure the absorbance. LFT 600 EC freeze dryer was used to obtain the freeze dried powder of the poly herbal drug. Cells were incubated at 37 °C in a humidified CO_2_ incubator (SHEL LAB/Sheldon manufacturing Inc. Cornelius, OR 97113, USA). Inverted fluorescence microscope (Olympus Optical Co. Ltd. 1X70-S1F2, Japan) for observation of cells, and photographs were taken using microscope digital camera (MDC200 2 M PIXELS, 2.0 USB). Deionized water from UV ultra-filtered water system (Waterproplus LABCONCO Corporation, Kansas city, Missouri 64132–2696) and distilled water was used in all experiments.

### Cell cultures

Human hepatocellular carcinoma cell line (HepG_2_) and human cervical adenocarcinoma cell line (HeLa) were cultured in Dulbecco’s Modified Eagle Medium (DMEM), supplemented with 10% heat inactivated fetal bovine serum (FBS), penicillin (100 U/mL) and streptomycin (100 U/mL). The cells were maintained in 25 cm^2^ plastic tissue culture flasks at 37 °C in a humidified atmosphere containing 5% CO_2_ in air. Exponentially growing cells were used in all experiments. The normal rat fibroblast (CC1) cell line was employed as the control. In all experiments cells were suspended in the growth medium and seeded in 24-well plates at 2 × 10^5^ cells/well. In all experiments negative control without LPG and positive control with cyclohexamide (50 μg/mL) were simultaneously conducted. The assays which needs cell lysates, the cell lysate was prepared by treating the cells with TritonX 100 (0.1%; 1 mL) and sonicating the contents for 20 s. The final suspension was centrifuged at 4000 rpm for 5 min for the removal of cell debris.

### Poly herbal drug and preparation of poly-herbal extract

The traditional poly herbal anti cancer drug Le Pana Guliya (LPG) was obtained from the traditional medicinal practitioner Dr. Mahinda Ekanayake (Reg. number: 11797), No: 9, Moragahapitiya, Balagola, Kengalle, Kandy, Sri Lanka. Sample of 5 g of LPG from three different batches soaked in distilled water (100 mL) was kept in the rotary shaker for 48 h in an air tight dark bottle. The extract was then filtered through a layer of muslin cloth and filtrate was centrifuged at 3000 rpm for 15 min at 4 °C to remove any debris.

The supernatant was freeze dried, and stored at −20 °C in an air tight vial until used. Each different extracts were used for all the assays carried out in this study.

The freeze dried extract was reconstituted with distilled water for experimental purposes.

The drug extracts were prepared in triplicate and each experiment was performed in triplicates to each preparation. Cell viability was determined as percentage of the absorbance of the treated cells to that of un-treated cells.

### Cell viability assay

The effect of aqueous extract of the LPG on the cell viability was determined by (3-(4,5-Dimethylthiazol-2-yl)-2,5-diphenyltetrazolium bromide (MTT) assay. The live cells reduce yellow MTT to purple formazan crystals by mitochondrial dehydrogenase enzyme [7]. The cells were seeded in 24 well plates (NUNC, Denmark) and cultured over-night as mentioned above. The mono-layers of cells were treated with different concentrations of LPG extracts prepared in culture medium and incubated in a CO_2_ incubator at 37 °C for 24 h.

After 24 h, the growth medium was replaced with 1.0 mL of minimum essential media (MEM), and 100 µL of MTT (5 mg/mL in PBS). The cells were incubated at 37 °C for 4 h and the medium was carefully removed. The formazan product was dissolved in acidified isopropanol (0.05 M HCl in Isopropyl alcohol (IPA); 750 µL) and absorbance was read at 570 nm. Cell survival was expressed as a percentage of viable cells of treated samples to that of untreated cells (negative control).

### Lactate dehydrogenase (LDH) activity

Cytotoxicity induced by the drug assessed by lactate dehydrogenase (LDH) leakage into the culture medium was carried out with slight modifications as described in Fotakis and Timbrell 2006 [8]. Cells were seeded and treated as described in MTT assay. After 24 h incubation the culture medium was aspirated and centrifuged at 4000 rpm for 5 min and supernatant and the lysate were subjected to LDH assay using a commercially available, LDH assay kit (HUMAN).

The percentage LDH leakage to the medium was calculated using following equation.

$$\% \,{\text{LDH activity}} = \left[ {\left( {{{\text{Activity of the supernatant}} \mathord{\left/ {\vphantom {{\text{Activity of the supernatant}} {\text{Total activity}}}} \right. \kern-0pt} {\text{Total activity}}}} \right) \times 100} \right]$$

where total LDH activity = LDH activity of supernatant + LDH activity of the lysate.

### Estimation of protein content

The protein content of the cell lysate was determined described by Lowry et al. 1951 [9], after treatment with LPG for 24 h. Briefly, sodium hydroxide (2 M, 100 μL) was added to the cell lysate (100 μL) and the mixture was incubated at 100 °C for 10 min. A mixture (1 mL) prepared by dilution (100:1:1) with Na_2_CO_3_ (2%), CuSO_4_·5H_2_O (1%) and sodium potassium tartrate (2%) was then added to the test solution and Folin–Ciocalteu reagent was added after 10 min. the samples were incubated for 30 min at room temperature in the dark. The absorbance was measured at 750 nm. Bovine serum albumin (BSA) was used for the calibration curve to determine the protein content of cell lysate. The percentage protein content of the treated cells to that of untreated cells was calculated using following equation.

$$\% \,{\text{of}}\,{\text{protein}}\,{\text{content}} = \left[ {{{{\text{Protein}}\,{\text{content}}\,{\text{of}}\,{\text{treated sample}}} \mathord{\left/ {\vphantom {{{\text{Protein}}\,{\text{content}}\,{\text{of}}\,{\text{treated sample}}} {\text{Protein content of the untreated}}}} \right. \kern-0pt} {\text{Protein content of the untreated}}}} \right] \times 100$$

### Light microscopy

HepG_2_, HeLa and CC1 cells at 70% confluence were treated with different concentrations of drug extracts for 24 h and observed under phase-contrast inverted fluorescence microscope (40×). The changes in morphology were compared with positive and negative controls.

### Griess nitrite assay

The cell supernatant was used to assay nitric oxide production in cells, as explained by the method of Green et al. 1982 [10]. Briefly 100 μL of the culture supernatant was incubated with 100 μL of Griess reagent (1% sulphanilamide in 0.1 mol/l HCl and 0.1% *N*-(1-naphthyl) ethylenediaminedihydrochloride at room temperature for 10 min.

The absorbance was measured at 540 nm. The nitrite content was calculated based on a standard curve constructed with NaNO_2_ and the nitrite content is expressed as nmoles.

### Determination of cellular reduced glutathione (GSH) levels and effect of endogenous GSH on the cell viability LPG

The total reduced glutathione (GSH) content of the HepG_2_, HeLa and CC1 cells were determined using the methods described by in Padma et al. 2007 [11] with slight modifications. The effect of exogenous GSH on the cell viability was also investigated in the presence of LPG. Briefly the cells were seeded as described earlier. The effect of endogenous GSH on cell viability was also determined after addition of GSH (25 μg mL) in the presence of LPG at same concentrations of EC_50_ obtained for MTT assays for respective cell lines. Negative control for each cell line was also carried out simultaneously. The cell viability was determined by MTT assay as described earlier. The GSH content was calculated based on a standard curve constructed with a series of reduced glutathione standards (0.5–3 µg/mL).

### Measurement of mitochondrial membrane potential (MMP)

Rhodamine 123 was used to evaluate the changes in mitochondrial membrane potential as described previously [12]. Briefly cells were incubated with LPG for 24 h. Cells were then washed with PBS (pH 7.4) and fixed with 70% ice cold ethanol.

Rhodamine 123 (20 μL; 10 μg/mL) was added to each well and incubated in the dark at 37 °C for 30 min. The cells were then washed gently with ice cold PBS twice and examined immediately using phase-contrast inverted fluorescence microscope (40×).

### Caspase 3 activity

Caspase-3 activity of HepG_2_ and HeLa was assayed and compared with normal cells (CC1) according to the manufacturer’s instructions of Caspase-3/CPP 32 Colorimetric Assay Kit. Briefly the cells were seeded in a 12-well plate with a density of 2 × 10^6^ cells/well, and treated with different concentrations of LPG in triplicates.

### Ethidium bromide and acridine orange staining

Ethidium bromide and acridine orange staining was carried out to determine the induction of apoptosis by LPG according to the method described by Ribble et al. and Soysa et al. [13, 14] with slight modifications. Cells were seeded in 12 well plates and the confluent layer was treated with LPG at different concentrations for 24 h as described previously. The adherent cells were washed carefully with 1.0 mL of PBS followed by addition of 20 μL of the dye mix containing ethidium bromide (100 mg/mL) and acridine orange (100 mg/mL). Morphological changes were examined immediately using phase-contrast inverted fluorescence microscope (40×) under UV lamp. Live cells with normal nuclear chromatin exhibited green nuclear staining and the cells undergoing apoptosis showed orange to red [14]. The changes in morphology were compared with positive and negative controls. Images were photographed using digital imaging system connected to microscope.

### DNA fragmentation assay

The isolation of fragmented DNA was carried out according to the procedure of Kasibhatla et al. [15] with slight modifications.

Briefly, cells (2 × 10^6^) were seeded in 12 well plates and treated with different concentrations (i.e. 0.5–2.5 for HepG_2_, 2.5–20.0 for HeLa and 50.0–500 μg/mL) of LPG for 24 h respectively. The cells were washed with PBS and trypsinized. The cell pellets were incubated with 20 μL lysis buffer (10 mM EDTA, 50 mMTris-HCl, 0.5% Sodium lauryl sarcosinate;pH 8) and 10 μL RNase A (final concentration 500 U/mL) at 37 °C for 4 h followed by digestion with proteinaseK for overnight at 50 °C. The samples were mixed with 8 μL of 6× DNA loading buffer. The DNA samples were subjected to electrophoresis on agarose gel (1.5%) in TBE buffer (89 mMTris-HCl, 89 mM Boric acid, 2 mM EDTA, pH 8.4) containing ethidium bromide (0.5 μg/mL The gel was run at 45 V and DNA was photographed using a UVI pro gel documentation system (UVItec UK.)

### Statistical analysis

The results were expressed as mean ± standard deviation (Mean ± SD). The measurements were performed in triplicate and values shown are representative for at least three independent experiments. Least square linear regression analysis was applied using Microsoft excel to determine the EC_50_ values and for the calibration curves. R^2^ > 0.99 was considered as linear for the calibration curves. Significant differences of each test result were statistically analyzed using “Mann–Whitney U” test significances with 95% significance using SPSS version 16.

## Results and discussion

MTT assay is a rapid colorimetric approach that widely used to determine cell growth and cell cytotoxicity. It measures mitochondrial activity through enzymatic reaction on the reduction of MTT to formazan [7].

**Fig. 1:**
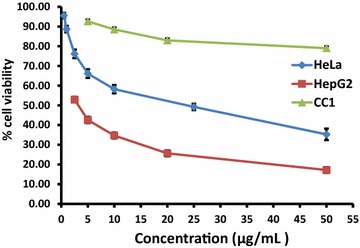
The percentage cell viability of on HepG_2_, HeLa and CC1 cell lines as determined by MTT assay after 24 h treatment with aqueous extract of the LPG. The data are presented as mean ± SD of six independent experiments for HepG_2_ and HeLa while nine independent experiments for CC1

The aqueous extract of LPG exhibited significant cytotoxicity (p < 0.05) against HepG_2_ and HeLa cells compared to CC1 cells as determined by MTT assay (Table 1; Fig. 1).

**Table 1:** EC_50_ values of MTT assay and LDH assay after 24 h incubation with LPG

Assay	EC_50_ (µg/mL) (n = 6)		
	HepG_2_	HeLa	CC1
MTT	2.72 ± 0.36*	19.03 ± 2.63**	213.07 ± 7.71
LDH	0.91 ± 0.03*	25.98 ± 0.59**	159.26 ± 3.09

*/** All results were mean n = 6 measurements ± standard deviation. “Mann–Whitney U” test at 95% confidence level showed a significant difference (p < 0.05) in both HepG_2_ and HeLa cells compared to CC1 cells in MTT and LDH assays

After 24 h incubation with cyclohexamide (Positive control) at a concentration of 50 µg/mL, the tested cells showed percentage viability of 64.43 ± 3.01% for HepG_2_, 75.66 ± 1.06% for HeLa and 71.93 ± 2.66% for CC1 cells. In contrast, the percentage viability obtained for MTT assay at the same concentration of LPG (50 µg/mL) treatment was 17.2 ± 1.47%, 35.26 ± 2.9% and 79.01 ± 1.59% for HepG_2_, HeLa and CC1 cells respectively. The EC_50_ obtained for MTT indicate that the cytotoxicity of the crude extract against the cancer cell lines is within the limit of cytotoxicity (EC_50_ < 30 µg/mL), as reported by the American National Cancer Institute (NCI) over 72 h post exposure and it is beyond the limits for CC1 cells [16].

Leakage of cytoplasmic located enzyme LDH into the extracellular medium is measured in lactate dehydrogenase (LDH) assay. Previous studies suggest that LDH is a more reliable and accurate marker of cytotoxicity, since damaged cells is fragmented completely during the course of prolonged incubation with substances [17]. Xia et al. reported that the intracellular LDH release to the medium is a measure of irreversible cell death due to cell membrane damage, where it is directly up regulate the subsequent induction of apoptosis [18].

**Fig. 2:**
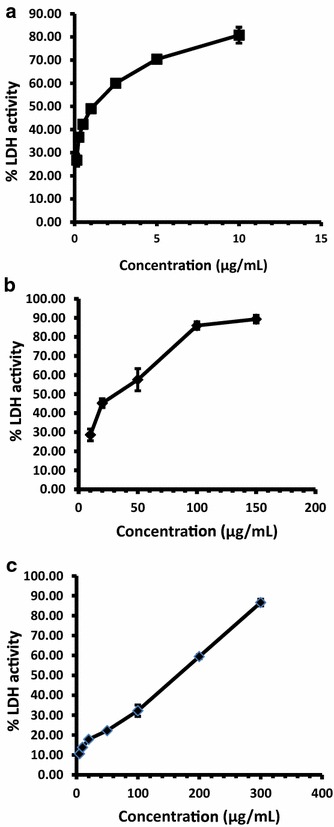
Dose dependent % LDH activity after incubation with LPG for 24 h. **a** HepG_2_ cells; **b** HeLa cells and **c** CC1 cells. The data are presented as mean ± SD of six independent experiments

In the present study there was a dose dependent increase in the LDH release observed at increasing concentrations of LPG (Table 1; Fig. 2) in all tested cell types. The percentage LDH release in untreated HepG_2_, HeLa and CC1 cells were 14.48 ± 1.62%, 6.9 ± 0.34% and 8.57 ± 2.02% respectively. Present study further shows that LPG exerts a high cytotoxicity against cancer cells investigated but not in normal CC1 cells.

It has been identified that the cellular stress conditions interfere with signaling pathways in protein synthesis [19]. Protein content in the lysate was determined in all three cell types after 24 h exposure of LPG. The results showed that there was a decrease in total protein content inHepG_2_ and HeLa cells treated with LPG compared to untreated cells.

**Fig. 3:**
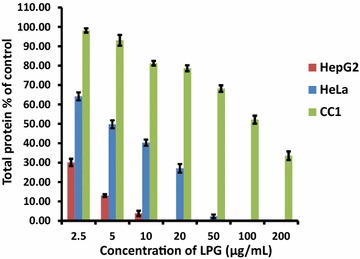
Effect of LPG at different concentrations on total protein present in HepG_2_, HeLa and CC1 cell lysate. Data are present as mean ± SD from three independent experiments

However, CC1 cells contain more than 80% of protein compared to that of untreated cells at concentrations between 2.5 and 10 μg/mL of LPG (Fig. 3). This result indicates that the LPG induces an inhibitory mechanism of protein synthesis in cancer cells we investigated causing cell death.

**Fig. 4:**
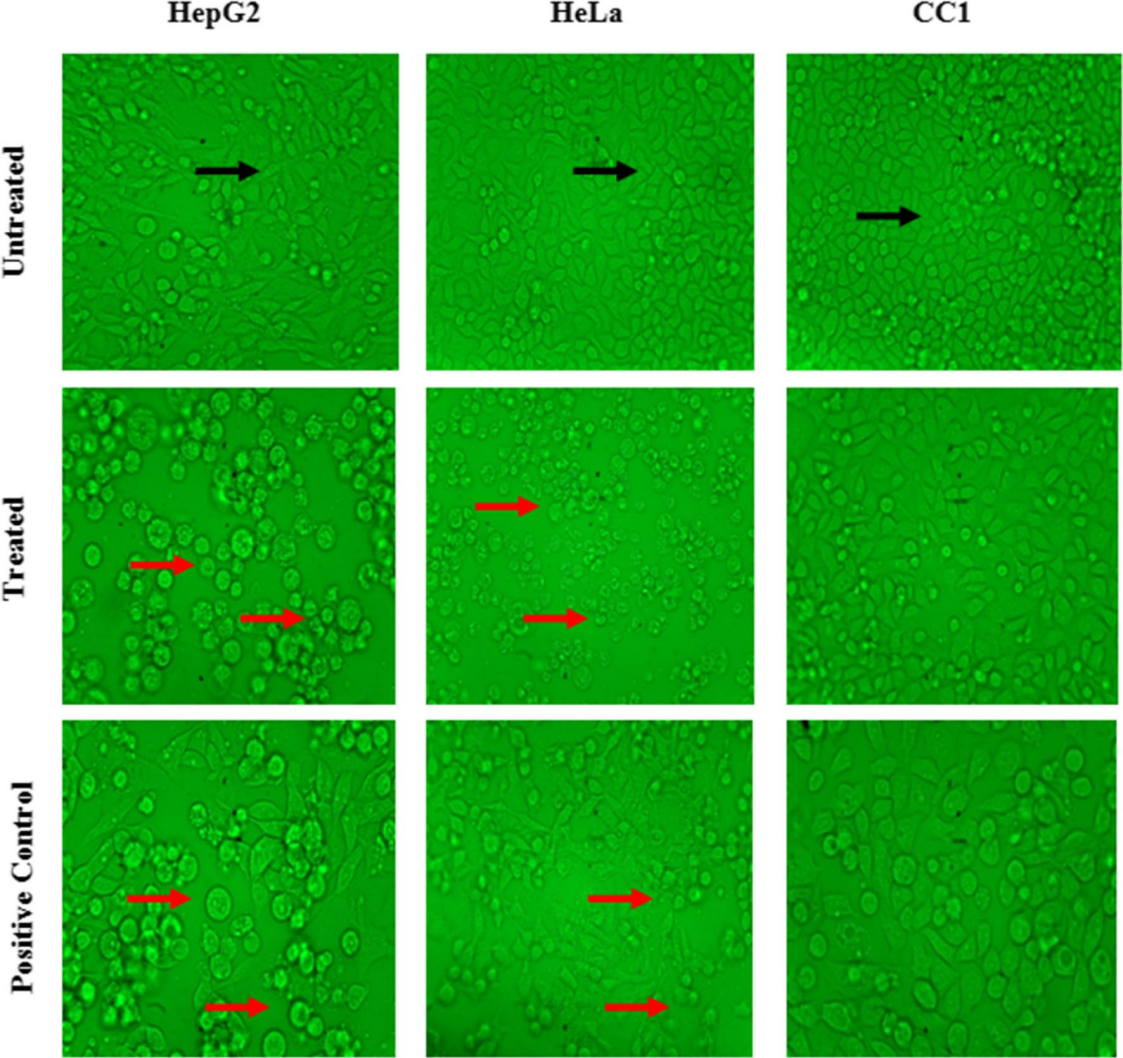
Light microscopy images of HepG_2_, HeLa and CC1 cells treated with their respective EC50 values, with magnification of 40×. (*Black arrow* indicates healthy spindle shape cells; *Red arrow* dead and shrinkage cells due to the LPG treatment)

The cytoplasmic condensation, cell shrinkage and condensation and aggregation of the nuclear chromatin, loss of contact with neighbouring cells, signs of membrane blebbing characteristic to apoptosis were observed in HepG_2_ and HeLa cells treated with LPG [20]. The untreated cells (negative control) of HepG_2_ and HeLa cells show a normal morphology (Spindle shape/elongated cells) that adhered to the culture plate with no or minimum number of cell death In contrast to the HepG_2_ and HeLa cells, the CC1 cells does not show any significant morphological changes even at a concentration of 500 µg/mL (Fig. 4).

**Fig. 5:**
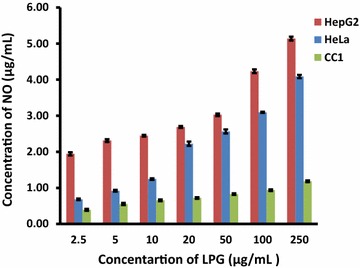
Effects of different concentrations of LPG on NO production in HepG_2_, HeLa and CC1 cells. Data are mean ± SD from three independent experiments performed in triplicates

It was reported that NO affects cellular decision of life and death either by turning on apoptotic pathways or by shutting them down [21, 22]. As NO is a highly reactive free radical within biological systems, it can react with biomolecules, molecular oxygen and heavy metals [22]. The supernatant collected from LPG treated cells was subjected to NO assay by Griess method. The LPG induced significant NO production (p < 0.05) in treated cells compared to the untreated cells as well as with compared to treated CC1 cells in HepG2 and HeLa cells. The CC1also shows an increase level of NO production with the concentration of the LPG compared to its untreated cells in dose dependent manner (Fig. 5).

Previous reports showed that an excessive and unregulated NO synthesis has been implicated to regression of tumorgenicity and metastasis of tumor cells via alterations of the expression of apoptosis associated proteins [23]. The elevated NO levels by LPG inhibit cell proliferation and trigger apoptosis [24]. This is through DNA damage caused by DNA modification or DNA strand breakage ultimately leading to apoptosis [25].

Therefore, it is evident that generated nitrite from NO has played a significant role in inhibition of HepG_2_ and HeLa cell growth.

One of the most complex aspects in the regulation of cell death is the role of intracellular oxidation of biomolecules including proteins. It was initially proposed that cellular oxidative stress could be a general mediator of apoptosis [26]. In fact, exposure to reactive oxygen and nitrogen species (RONS)such as hydrogen peroxide (H_2_O_2_) or nitric oxide (NO) induces cell death via apoptosis in different cell types [27]. It was reported that the direct oxidant treatment and deregulated intracellular production of ROS are equally harmful to the cell and are countered by various antioxidant defenses. Among them, the tri-peptide GSH is the most rapid and abundant weapon against ROS and regulates the redox state of many other cellular constituents [26].

**Fig. 6:**
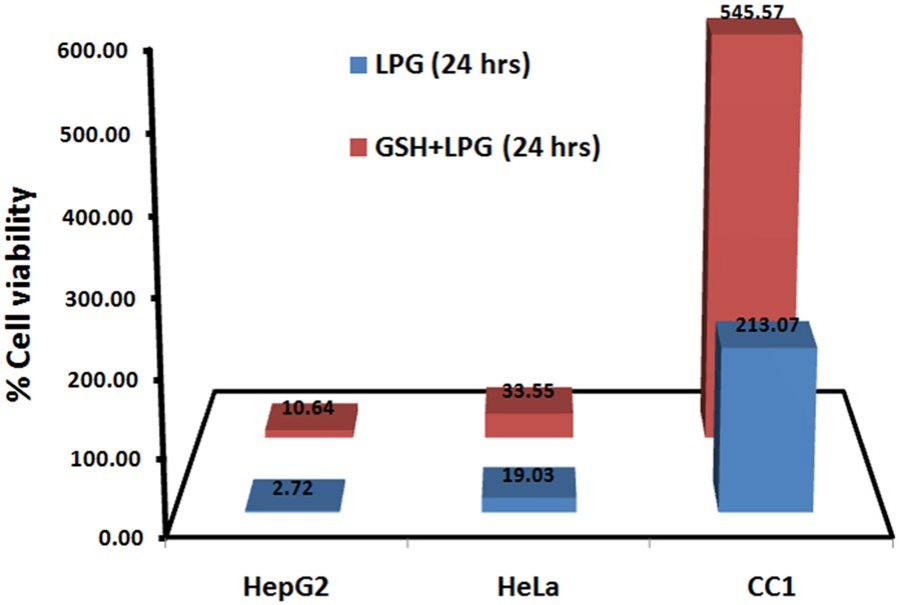
Effect of exogenous GSH on EC_50_values of the HepG_2_, HeLa and CC1in the presence and absence of exogenous GSH

GSH plays an important role in protection of cells against oxidative stress [11]. It has been reported that, cellular glutathione level is an important determinant for the activity of anti-cancer agents [11]. Increase in GSH levels and the activity of its related enzymes have been characterized as one of the factors, which could contribute to the tumor resistant to either radiotherapy or chemotherapy. Depletion of intracellular GSH is an early hallmark in the onset of apoptosis [28]. The intracellular GSH depletion might be resulted either from increased intracellular oxidation of GSH or stimulated GSH extrusion through a specific carrier or the inhibition of GSH synthesis or the direct conjugation of GSH with drug [28]. GSH levels were depleted significantly (p < 0.05) after treatment with LPG in all three cell lines but more effective in HepG_2_ and HeLa cells (Table 2). Furthermore in the presence of exogenous GSH (25 μg mL^−1^) we observed that the cell viability of tested cancer cells as well as control CC1 cells has increased (p < 0.05) compared to the GSH untreated cells. The increase is more prominent in HepG_2_ cells (Fig. 6).

**Table 2:** Effect of LPG on GSH levels in HepG_2_, HeLa and CC1 cells after 24 h treatment

Cells	Total GSH content (μg/mL)			
	Control	Treated (EC_50_)	+ve control	% Reduction
HepG2	10.15 ± 0.26	3.51 ± 0.23	5.72 ± 0.51	65.42
Hela	7.11 ± 0.11	2.22 ± 0.18	3.45 ± 0.32	68.78
CC1	9.41 ± 0.28	7.02 ± 0.75	6.06 ± 0.81	25.61

Data are mean ± SD from three independent experiments performed in triplicates

The results suggest that, depletion of GSH by LPG may contribute to the accumulation of RONS in the cells producing redox imbalance of the cells. This in turn leads to oxidation of biomolecules which are vital for cellular functions and membrane integrity causing cell death through stimulating of downstream events of apoptosis. Furthermore, reversing the cell death via neutralizing of ROS by exogenous GSH in the presence of LPG confirms that the induction of cell death is caused by oxidative stress.

The light microscopic photographs upon the treatment with LPG indicate prominent features of apoptosis. Similarly in the presence of high levels of NO and depletion in cellular GSH suggested that the LPG may exert its cytotoxicity via induction of apoptotic pathway.

**Fig. 7:**
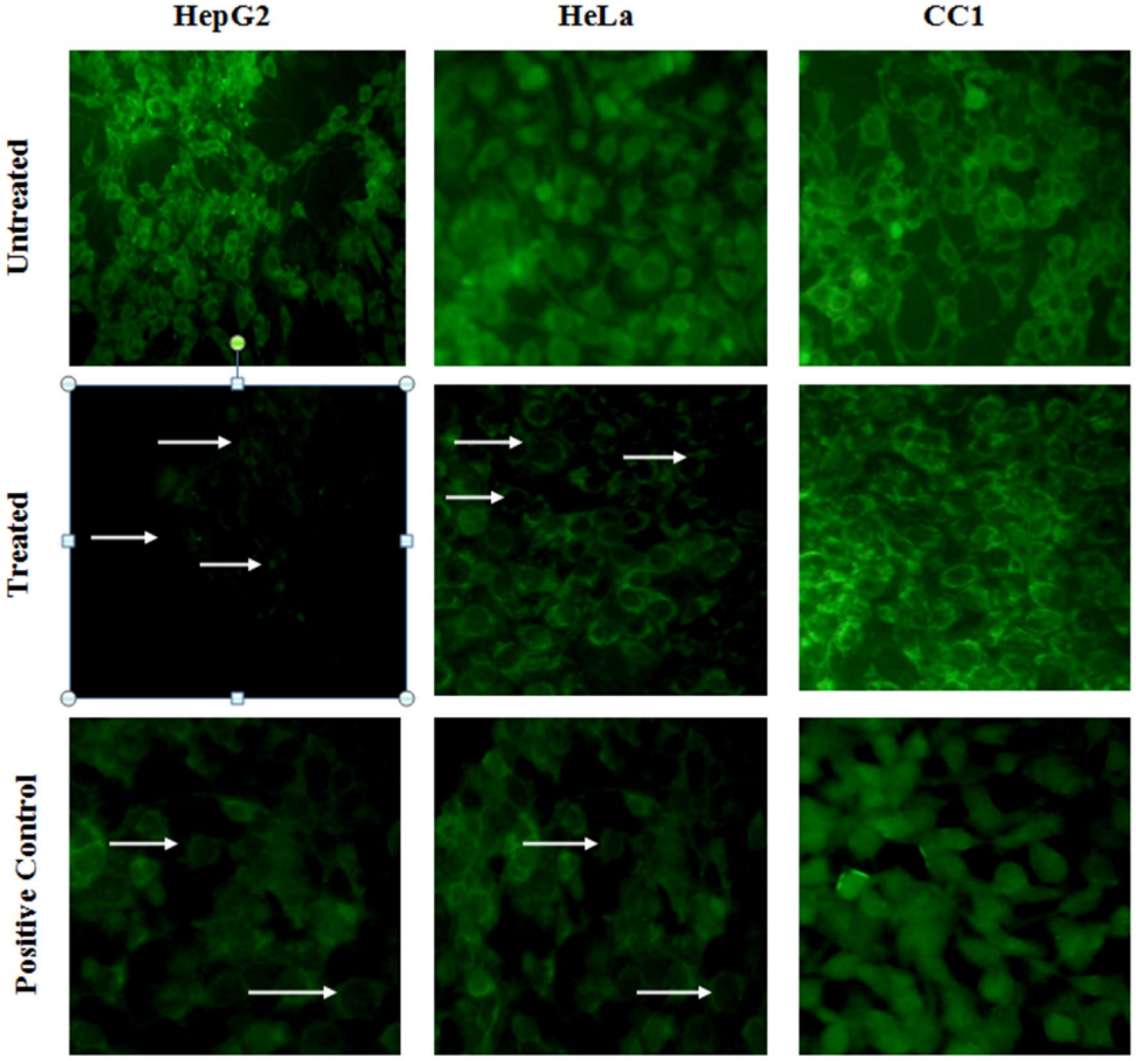
Mitochondrial staining using rhodamine 123 of HepG_2_, HeLa and CC1 in the presence or absence of the LPG. Cells treated with EC50 dose of the LPG for 24 h showing decreased membrane potential as indicated by the *arrows*. (Original magnification of 40×)

Depolarization in mitochondrial membrane potential (MMP/∆ψ_m_) is a characteristic feature of apoptosis. Excessive intra cellular ROS production has been shown to induce apoptosis by disrupting MMP [29, 30]. Mitochondrial membrane potential was evaluated by staining with rhodamine 123. Green fluorescence is observed in cells with high membrane potential. LPG was able to decrease the mitochondrial membrane potential in both HepG_2_ and HeLa cells. Untreated cells in each cell type which are in live state showed high uptake of fluorescent dyes (Fig. 7). There was no prominent change in fluorescence intensity in CC1 cells.

Changes in the ∆ψ_m_ have been originally hypothesized to be early, coerce events in the apoptotic signaling pathway [31]. It was reported that the mitochondrial permeability transition pore (PTP) which act as the “mega-channel” that results in the release of certain mitochondrial apoptogenic factors in some cell types during apoptosis [32]. LPG results in opening up of PTP leading to activation of caspase-3 through cascade of intracellular events resulting cell membrane blebbing, nuclear condensation and DNA fragmentation as a results of depletion of ∆ψ_m_.

**Fig. 8:**
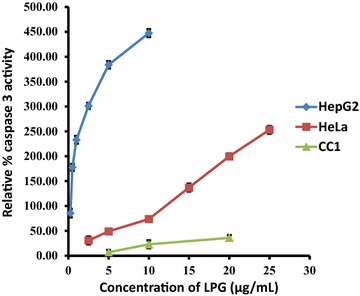
Total caspase three activities of HepG_2_, HeLa and CC1 cells obtained after the treatment with various concentrations of LPG for 24 h. Data are mean ± SD from three independent experiments performed in triplicates

We examined the effect of LPG on the cascade of caspases that are crucial initiators or effectors in the cell death pathways. Enzymatic activity of caspase-3 was determined after 24 h of incubation. A prominent activation of caspase-3 occurred even at very low concentrations of LPG in cancer cells after 24 h of incubation (Fig. 8) indicating that LPG induces the apoptotic cell death pathway compared to control CC1 cells.

Induction of apoptosis in HepG_2_ and HeLa cells by LPG were observed in the presence of AO/EB staining. Acridine orange (AO) permeates both live and dead cells and stains DNA and makes the nucleus appear green while ethidium bromide (EB) is only taken up by cells with damaged cell membranes [14]. Thus, live cells will be uniformly stained green, apoptotic cells will be stained as orange or displayed orange fragments, nuclear fragmentation, presence of apoptotic bodies and blebbing when observed under fluorescence microscope depending on the degree of loss of membrane integrity.

**Fig. 9:**
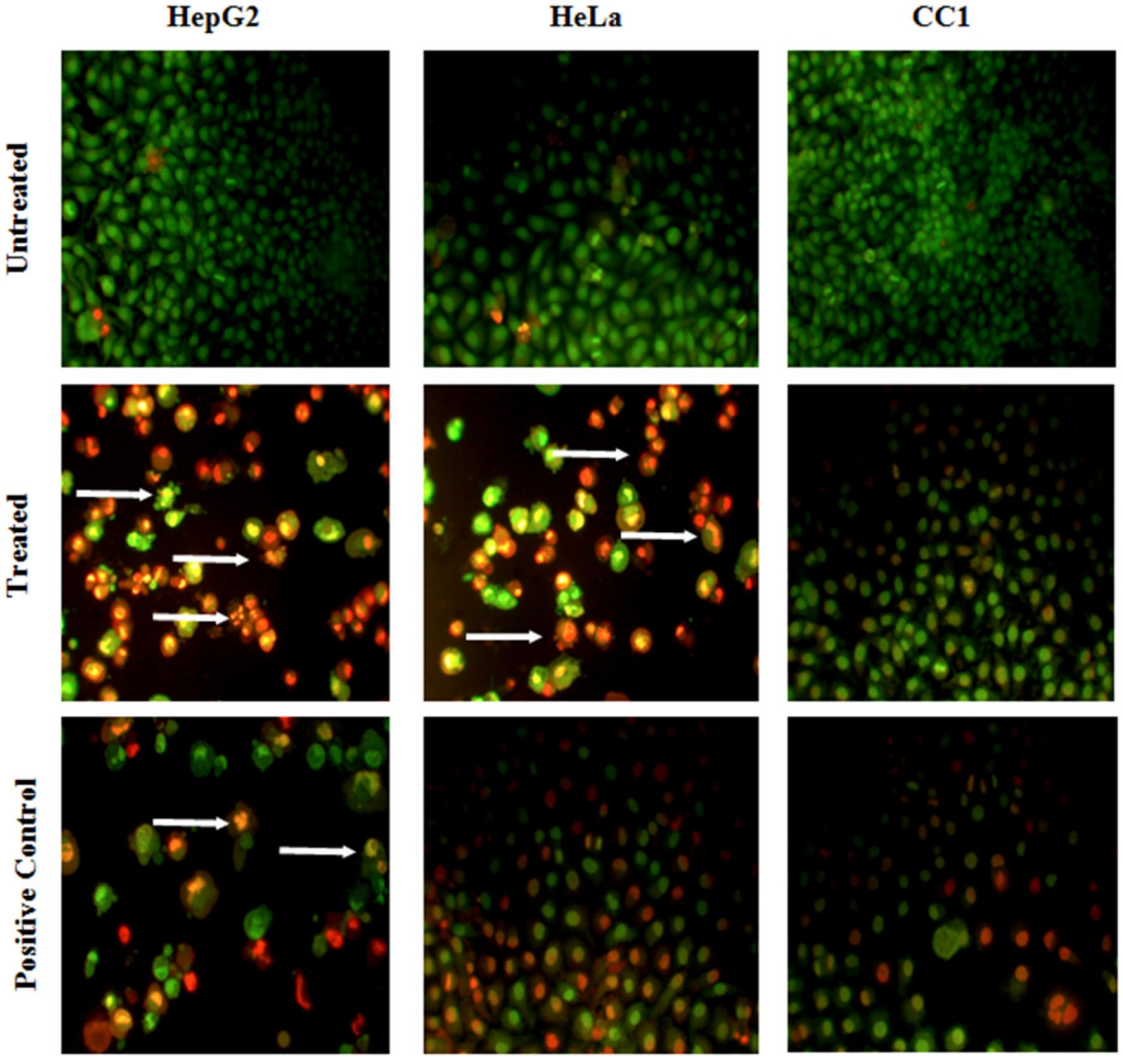
Apoptotic morphology detection by Acridine orange-ethidium bromide (AO/EB) fluorescent staining of HepG_2_, HeLa and CC1 cell lines treated with the LPG for 24 h. *Arrows* indicates the characteristic morphological features of apoptosis fragmented nuclei, cytoplasmic blebbing (Original magnification of 40×)

Following acridine orange and ethidium bromide staining, cells treated with LPG caused typical apoptotic morphological changes including chromatin condensation, membrane blebbing, and fragmented nuclei in HepG_2_ and HeLa cells contrast to the controls (untreated cells). The CC1 cells does not show any signs of apoptosis even at high concentrations (500 µg/mL) (Fig. 9).

This was further confirmed by the DNA fragmentation assay indicating a unique ladder banding pattern. With the activation of Caspase-3 it cleaves inhibitor of caspase activated DNase (ICAD), promoting release of active caspase-activated DNase (CAD) [33], The activated CAD then cleaves oligonucleosomal DNA at the inter-nucleosomal linker sites yielding DNA fragments in multiples of 180 base pairs [34].

**Fig. 10:**
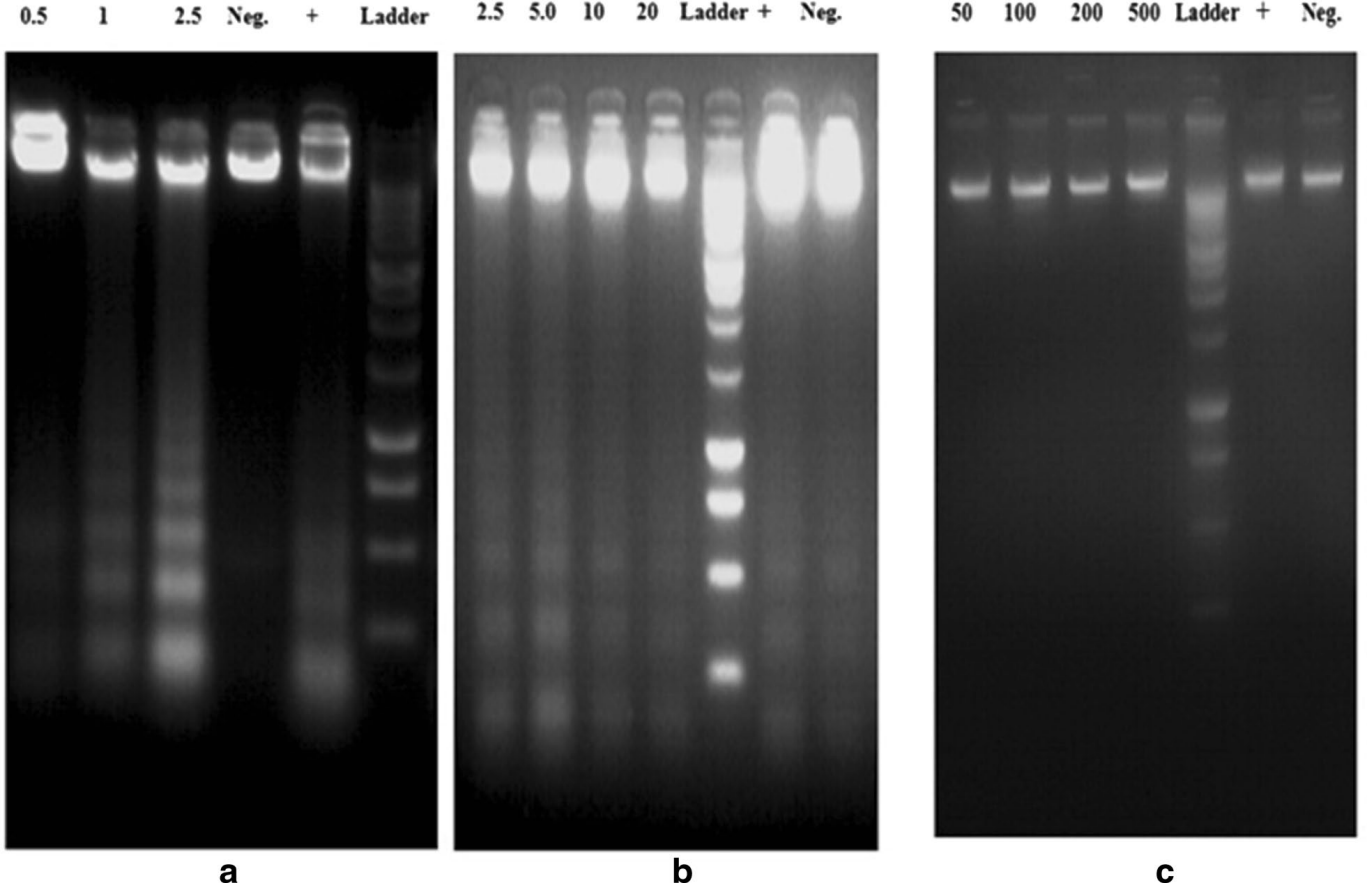
Agarose gel electrophoresis of DNA after treatment of LPG; **a** HepG_2_ cells, **b** HeLa cells and **c** CC1 cells treated with different concentrations of LPG (all concentrations are in μg/mL). The concentration of the Positive control cyclohexamide in all three cell types was 50 μg/mL

DNA fragmentation was observed in HepG_2_ and HeLa cells which exposed to the LPG for 24 h. Un-treated control cells showed no evidence of DNA fragmentation in CC1 cells even at high concentrations (100 µg/mL) of LPG for 24 h. The positive control cyclohexamide at 50 μg/mL has induced DNA fragmentation in both HepG_2_ and HeLa cells (Fig. 10).

## Conclusion

Based on the results we can conclude that LPG stimulates tumor specific oxidative stress. Activation of caspases through mitochondrial impairment caused by oxidative stress stimulates downstream events of apoptosis leading to DNA fragmentation and cell death in HepG2 and HeLa cells. LPG is more effective in inducing apoptosis in HepG_2_ cells and minimal cytotoxicity towards normal cell line CC1. The potent anticancer and apoptotic effects of LPG observed in the present study provide the scientific proof of the traditional knowledge in using LPG as an anticancer agent.
